# Analytical and Sample Preparation Protocol for Therapeutic Drug Monitoring of 12 Thiopurine Metabolites Related to Clinical Treatment of Inflammatory Bowel Disease

**DOI:** 10.3390/molecules23071744

**Published:** 2018-07-17

**Authors:** Daniel Pecher, Svetlana Dokupilová, Zuzana Zelinková, Maikel Peppelenbosch, Jana Lučeničová, Veronika Mikušová, Peter Mikuš

**Affiliations:** 1 Department of Pharmaceutical Analysis and Nuclear Pharmacy, Faculty of Pharmacy, Comenius University in Bratislava, Odbojárov 10, SK-832 32 Bratislava, Slovakia; pecher1@uniba.sk (D.P.); dokupilova@fpharm.uniba.sk (S.D.); 2 Toxicological and Antidoping Center, Faculty of Pharmacy, Comenius University in Bratislava, Odbojárov 10, SK-832 32 Bratislava, Slovakia; 3 Department of Gastroenterology, St Michael’s Hospital, Satinského 1, SK-811 08 Bratislava, Slovakia; zuzana.zelinkova@nsmas.sk; 4 Gastrolab, Erasmus Medical Center, Wytemaweg 80, 3015 CN Rotterdam, The Netherlands; m.peppelenbosch@erasmusmc.nl; 5 Department of Biochemistry & Hematology, St Michael’s Hospital, Satinského 1, SK-811 08 Bratislava, Slovakia; jana.lucenicova@nsmas.sk; 6 Department of Galenic Pharmacy, Faculty of Pharmacy, Comenius University in Bratislava, Odbojárov 10, SK-832 32 Bratislava, Slovakia; janosova@fpharm.uniba.sk

**Keywords:** inflammatory bowel diseases, therapeutic drug monitoring, profiling of thiopurines, comprehensive stability study, sample treatment, clinical analysis, high performance liquid chromatography, mass spectrometry

## Abstract

Thiopurines (TP) represent an important therapeutic tool for the treatment of inflammatory bowel diseases (IBD) in the current situation of rising incidence and health care costs. The results of multiple clinical studies aimed at finding correlations between levels of TP metabolites and response of IBD patients to the treatment are, however, often controversial due to variability in analytical and sample preparation procedures among these studies. In this work, therefore, an updated analytical and sample preparation procedure for therapeutic drug monitoring (TDM) of TP metabolites in blood samples obtained from patients with IBD was proposed to establish a unified protocol. An advanced analytical method based on ion-exchange liquid chromatography hyphenated with tandem mass spectrometry (IEC-ESI-MS/MS) was used for the determination of the profiles of 12 individual TP metabolites in the particular steps of sample preparation procedure including blood collection, red blood cells (RBC) isolation, lysis, and storage. Favorable performance parameters of the IEC-ESI-MS/MS method (LLOQs 1–10 nmol/L, accuracy 95–105%, intra-day and inter-day precision < 10%, selectivity demonstrated via no sample matrix interferences) and acceptable stability (peak area fluctuations < 15%) of clinical samples under the proposed sample preparation conditions {(i) EDTA anticoagulant tube for the blood collection; (ii) 4 °C and 4 h between the sample collection and RBC isolation; (iii) phosphate-buffered saline for RBC washing and re-suspendation; (iv) −20 °C for RBC lysis and short-term storage; (v) 50 mmol/L phosphate buffer, pH 7.4, 10 mmol/L DTT as a stabilizing medium for TPN in RBC lysates} demonstrated the suitability of such protocol for a well-defined and reliable routine use in studies on thiopurines TDM.

## 1. Introduction

**Figure 1:**
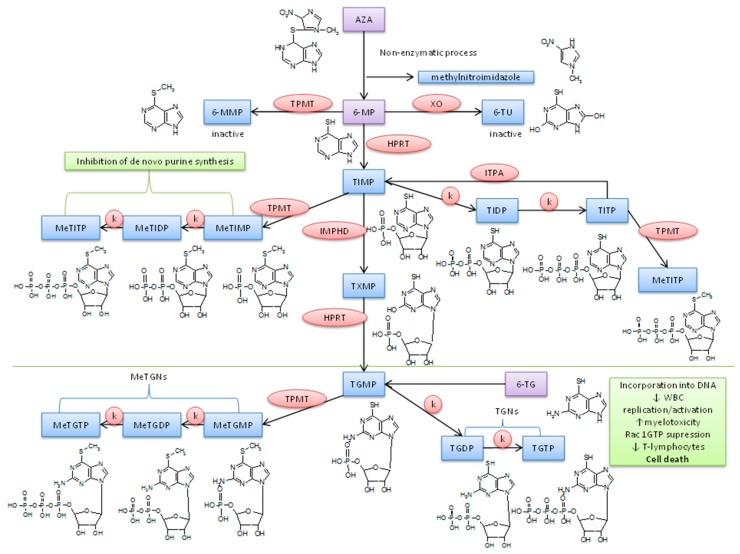
Scheme of thiopurine metabolism. AZA, azathioprine; 6-MMP, 6-methylmercaptopurine; TPMT, thiopurine S-methyltransferase; 6-MP, 6-mercaptopurine; XO, xanthine oxidase; 6-TU, 6-thiouric acid; HPRT, hypoxanthine guanine phosphoribosyltransferase; TIMP, 6-thioinosine 5′-monophosphate; TIDP, 6-thioinosine 5′-diphosphate; TITP, 6-thioinosine 5′-triphosphate; ITPA, inosine triphosphate pyrophosphatase; MeTIMP, 6-methylthioinosine 5′-monophosphate; MeTIDP, 6-methylthioinosine 5′-diphosphate; MeTITP, 6-methyl-thioinosine 5′-triphosphate; IMPHD, inosine monophosphate dehydrogenase; TXMP, 6-thioxanthine 5′-mono-phosphate; TGMP, 6-thioguanosine 5′-monophosphate; TGDP, 6-thioguanosine 5′-diphosphate; TGTP, 6-thio-guanosine 5′-triphosphate; 6-TG, 6-thioguanine; MeTGMP, 6-methylthioguanosine 5′-monophosphate; MeTGDP, 6-methylthioguanosine 5′-diphosphate; MeTGTP, 6-methylthioguanosine 5′-triphosphate.

Inflammatory bowel diseases (IBD), namely Crohn’s disease (CD) and ulcerative colitis (UC), are autoimmune disorders of unclear etiology with the characteristic intermittent course, which are manifested by chronic gastrointestinal inflammation [1]. The disease affects typically young people at the age between 20 and 40 and its incidence is rising [2,3]. Thiopurines (TP), namely azathioprine (AZA), 6-thioguanine (6-TG) and 6-mercaptopurine (6-MP), are effective in the maintenance of remission of IBD [4,5,6,7] and in the era of new costly treatment modalities for IBD still represent pharmacoeconomically relevant therapeutic option. Their use is, however, limited by the side effects that occur in up to one third of patients [8,9,10,11] and the underlying mechanism of these side effects is not fully understood. TP are so-called “pro-drugs” and their effect is preceded by a complex biotransformation (see Figure 1). 6-thioguanine 5′-triphosphate (TGTP) is considered as a main active metabolite. It is involved in the control of T-cell apoptosis by the modulation of Rac1 activation upon CD28 co-stimulation. TGTP binds to Rac1 instead of guanosine triphosphate which leads to the suppression of Rac 1 target genes, e.g., mitogen-activated protein kinase, NF-kappaB, and bcl-x(L), and, therefore, to the mitochondrial pathway of apoptosis [12]. Also, 6-methylthioinosine 5′-triphosphate (MeTIMP) can cause the inhibition of de novo purine synthesis [13]. Thus, TP seem to exert their immunosuppressive effects through at least two different metabolites interfering differentially with the immune response. The therapeutic effect of other individual AZA metabolites, is however unclear thus far.

Therapeutic drug monitoring (TDM) of the TP metabolites was suggested as a tool to improve the efficacy and safety of the treatment of IBD patients. Multiple clinical studies focusing at the role of TDM in optimizing TP treatment of IBD patients studied the associations between the levels of TP metabolites and the treatment efficacy and/or safety [14,15,16,17,18,19,20,21]. The results of these studies differ significantly contrasting strong associations of metabolic profiles with the treatment response and side effects of therapy with no association found. Putting aside the differences in design between the respective clinical studies, there are at least three main reasons, from the analytical point of view, causing the discrepant results, i.e., (i) what is analyzed; (ii) how the sample is prepared; (iii) how the sample is analyzed.

Firstly, the measured levels of the TP metabolites are usually represented as a sum (pool) of mono-, di- and tri-phosphate of individual ‘*class*’ of metabolites obtained after hydrolysis (dephosphorylation) of individual thiopurine nucleotides (TPN) (e.g., 6-thioguanine 5′-monophosphate (TGMP), 6-thioguanine 5′-diphosphate (TGDP) and TGTP are hydrolyzed to 6-TG, which amount is then determined). This could be insufficient in a case if individual TPN had different effects. By now, however, only three clinical studies, dealing with the therapeutic effect of individual TPN (ranging from 3 to 9 separated nucleotides), were published [22,23,24]. In one study, a significant correlation between TGDP/TGTP ratio and disease condition was observed. It was concluded that the TGDP levels higher than 15% of the total TPN could present a valuable parameter predicting a poor response to the TP treatment [22]. The other two studies, however, failed to confirm this correlation [23,24].

Secondly, the use of different protocols for the storage and preparation of the samples (blood, red blood cells (RBC)) obtained from the IBD patients can be another factor contributing to the discrepancies in the clinical results. Considering a limited stability and fast interconversion of the individual phosphated forms of TP, a proper protocol is of a high importance. There are two procedures currently used in the pre-analytic treatment of the samples (after thawing the RBC lysates until HPLC analysis) considered for the analysis of individual TPN. They were proposed by Rabel et al. [25] and Vikingsson et al. [26] and are based on ethylendiaminetetraacetic acid (EDTA) and phosphate buffer (PB), respectively, as the stabilizing agents for TPN. However, only limited data from the evaluation of the stability of individual TPN are available [26,27] and no comparison of these two procedures taking the full analytical profiles of individual TPN has been carried out so far. Moreover, other relevant sample preparation steps, such as the blood collection, RBC isolation, lysis and storage, are not unified in this field as well.

Thirdly, different analytical methods were applied for evaluating the individual TPN in blood samples. Vikingsson et al. [26] used a reversed-phase LC (RPLC) method with an ion-pairing agent and a UV and fluorescence detection for the separation of 9 TPN (6 methylated TPN and 3 thioguanine nucleotides oxidized to corresponding S-S products, i.e., with no need to stabilize the thiol groups in TPN). On the other hand, Hofmann et al. [27] directly determined 11 TPN metabolites using an ion exchange LC (IEC) with a mass spectrometry detection. As the method by Hofmann et al. [27] provided the most complex TPN profile published in the literature so far, it is worthy to use it for the evaluation and comparison of different established pre-analytic sample preparation procedures (i.e., [25] and [26]) as well as other relevant sample preparation steps applied in profiling individual TPN.

Hence, the aim of our work was to propose an effective protocol, integrating a powerful analytical method with an effective sample preparation procedure, applicable in current clinical practice dealing with the treatment of IBD patients by means of TP. It should serve for the advanced TDM based on a highly reliable determination of the individual TPN in clinical blood samples. For this purpose, particular parameters of the sample preparation procedure, including (i) type of anticoagulant used in the blood collection tubes (Li-Heparin vs. EDTA); (ii) time between the sample collection and RBC isolation (0–4 h); (iii) type of the RBC washing and re-suspendation (dilution) solution (0.9% saline vs. phosphate-buffered saline, PBS); (iv) temperature used for the RBC lysis and storage (−20 °C vs. −80 °C); (v) type of stabilizing agent (EDTA vs. PB) for TPN present in RBC lysates and analyzed samples, were comprehensively optimized with respect to achieving a maximum stability of the analyzed compounds. For the first time, an advanced IEC-ESI-MS/MS approach was applied to monitor the effect of such parameters on the complex TPN profiles. The proposed protocol should be useful in a well-defined profiling of the clinical samples obtained from the patients suffering from IBD.

## 2. Results and Discussion

**Figure 2:**
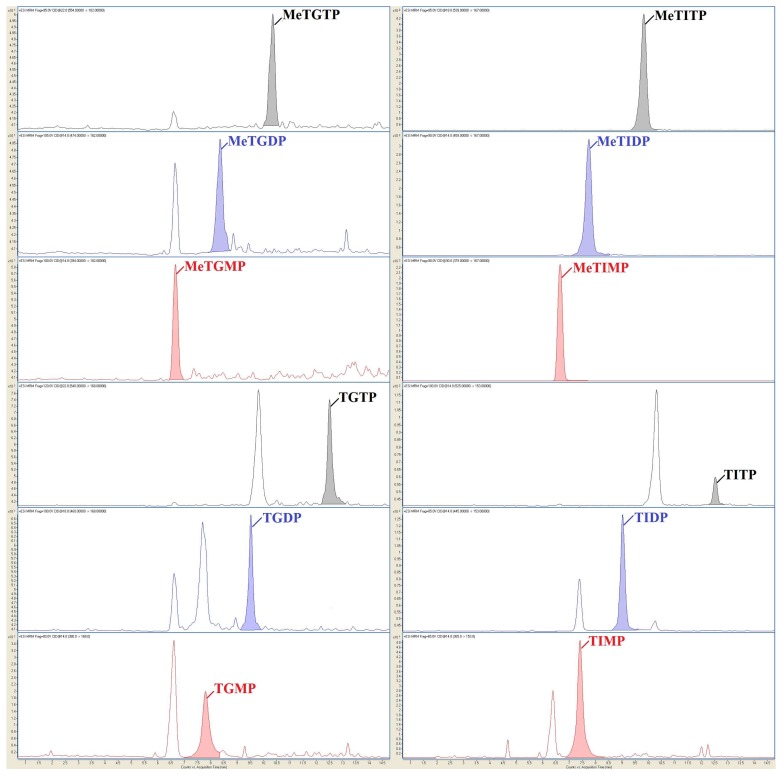
Representative profile of 12 thiopurine nucleotides in clinical sample taken from IBD patient treated by AZA. The RBC lysate sample was prepared according to the Section 3.3 and Section 3.4 with optimized sample preparation parameters (summarized in Section 4). The IEC-ESI-QqQ method with optimized operating parameters was used for the analysis (Section 3.5 and Section 3.6).

In our work, IEC-ESI-MS/MS method was used with two types of tandem mass spectrometry detection techniques, namely high-resolution Q-TOF (to confirm the identity of individual TPN via their exact molecular weights) and QqQ (to the sensitive determination of individual TPN). Then, the optimized and validated IEC-ESI-QqQ method served for the highly reliable and sensitive profiling of the clinical samples obtained from the patients suffering from IBD and treated by AZA, see representative profile in Figure 2. For the first time, the full TPN profiles (i.e., 12 TPN) could be considered in a comprehensive stability study testing the parameters important for the handling and treating of biological material in clinical practice. These parameters are discussed in the following subsections to see their experimental proof.

### 2.1. Effect of Stabilizing Agents for Phosphated Thiopurines

Considering the limited stability and fast interconversion of the individual phosphated forms of TP, proper chemical stabilization conditions are crucial for this type of analytes and must be considered at the beginning. The current pre-analytic treatments of RBC lysates for the individual TPN profiling are based on two main strategies [25,26]. In the first case [25] a 50 mmol/L EDTA solution with high pH (10.5) serves for the stabilization of TPN, while in the second case [26] a 50 mmol/L PB solution with physiological pH (7.4) serves for this purpose.

In our study, three sets of the standard solution mixtures including the mono-, di- and tri-phosphated TPN along with the corresponding (i.e., mono-, di- and tri-phosphated) internal standards in the first, second and third set, respectively, were prepared, each at two concentration levels of the analytes (5 µmol/L and 1 µmol/L) and with the addition of dithiothreitol (DTT; at a 1 mmol/L final concentration). Each of these mixtures was prepared in three different media, i.e., with EDTA and PB as stabilizing agents and in demineralized water (serving as a reference medium). The stability (i.e., differences in the intensity of the analytical signals) of TPN in such model samples was evaluated for six different time intervals of the sample storage, i.e., 0 (immediately after preparation), 12, 24, 48, 72 and 120 h. During these time intervals the prepared solutions were stored in the cooled compartment of the HPLC autosampler (temperature set at 4 °C). Each solution was measured three times at each time interval and the results were expressed as the ratios of the analytical signals obtained in the given time intervals (12; 24; 48; 72 or 120 h) to the reference time interval (0 h).

**Figure 3:**
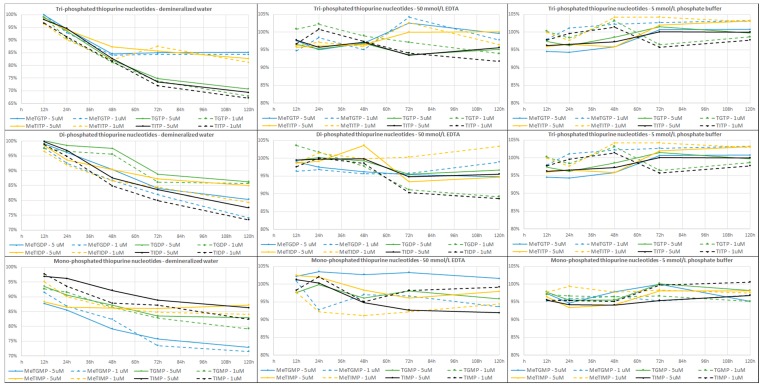
Stability of thiopurine nucleotides in different stabilization media during 5-day storage at 4 °C. Media used: demineralized water, serving as a reference medium (**left panels**), 50 mmol/L EDTA (**middle panels**), 5 mmol/L phosphate buffer (**right panels**). IEC-ESI-QqQ method (Section 3.5 and Section 3.6) was used for the analysis. For the sample preparation see Section 3. Materials and Methods.

The effect of the studied stabilizing agents on the TPN stability is illustrated in Figure 3. It is obvious from the left diagrams in Figure 3 that the pure demineralized water (i.e., without addition of the stabilizing agents) does not provide acceptable conditions for stabilizing TPN. A significant decrease in the concentrations of all the TPN with time is apparent. The concentrations of most of the TPN dropped below 90% of the starting value after 48 h of storage while they decreased down to 70% after 120 h. The obtained results confirmed a limited stability of the individual TPN even at 4 °C.

**Figure 4:**
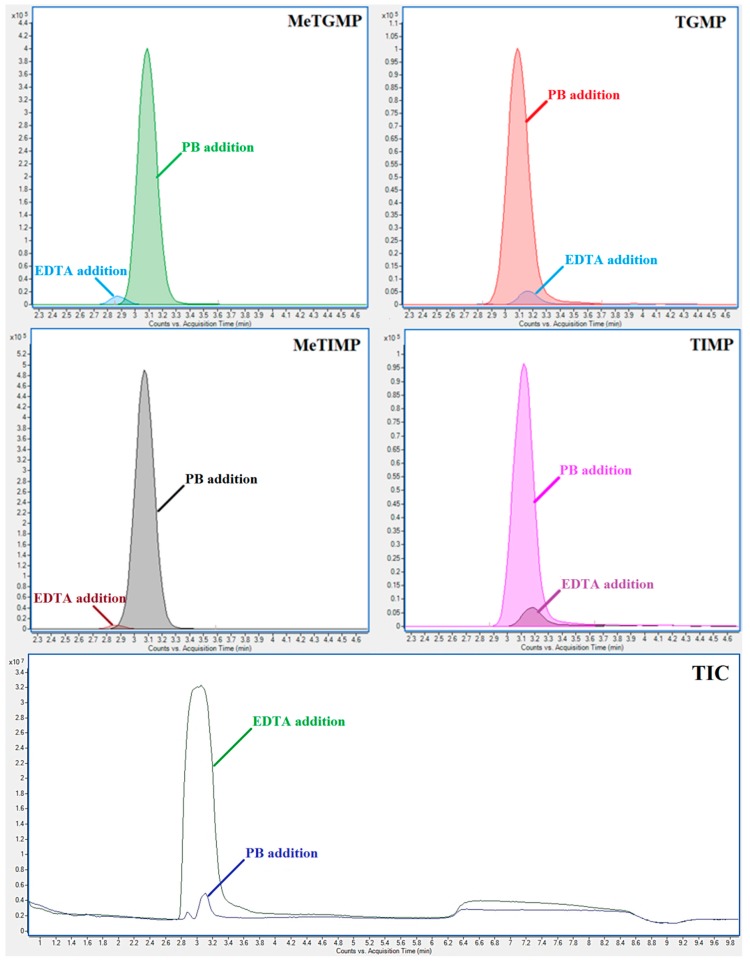
Influence of stabilization additive for phosphated thiopurines on detection signal. EIC chromatograms of MeTGMP; TGMP; MeTIMP and TIMP obtained with addition of EDTA and phosphate buffer (PB) as stabilizing agents. Standard solution of the same concentration was used for all analyses. Bottom panel: TIC chromatogram of the same sample. IEC-ESI-QqQ method (Section 3.5 and Section 3.6) was used for the analysis. For the sample preparation see Section 3. Materials and Methods.

A significant improvement of the TPN stability was observed with the addition of a 50 mmol/L EDTA solution (final concentration in the analyzed sample was 50 mmol/L EDTA). The concentrations of all the TPN are within a 90–105% interval of the initial values for all the tested time intervals (see the middle diagrams in Figure 3). However, significantly lower MS detection responses (i.e., absolute peak areas) of some TPN were obtained in comparison with those obtained in the pure demineralized water or in the PB solution. For example, the peak area of 6-methylthioinosine 5′-diphosphate (MeTIDP) in the EDTA solution represented only 19.63% of the peak area obtained in the PB solution. In some other cases (mainly monophosphates) the differences were even more pronounced: 6-methylthioguanine 5′-monophosphate (MeTGMP)—2.79%; TGMP—5.60%; MeTIMP—1.4%, and 6-thioinosine 5′-monophosphate (TIMP)—7.6%, when comparing the MS responses in the EDTA and PB solutions (see Figure 4). TGMP at the concentration level of 1 µmol/L was even below the limit of detection when using the EDTA medium. These signal suppressions most likely resulting from the matrix effect (such as the suppression of ionization in ESI) caused by the EDTA abundance and amplified by the similar elution properties of EDTA and monophosphated TP (see the lowest panel in Figure 4).

An addition of 50 mmol/L PB solution (final concentration in the analyzed sample was 5 mmol/L PB) provided the best results concerning both the stabilization of all the twelve TPN as well as the response of MS detection. The measured TPN concentrations ranged in a 95–105% interval of the initial values for all the tested time periods (see the right diagrams in Figure 3). Although a few values were outside of this interval they were still very close to the lower limit of 95%.

Based on these results, a 50 mmol/L PB solution with pH 7.4 and a 10 mmol/L DTT addition was proposed as the optimum-stabilizing medium for TPN, preferred when using the IEC-ESI-QqQ method for the analysis. In this way, the TPN samples should be stable (with the fluctuations of peak areas ca. ±5%) in the LC autosampler tempered at 4 °C for at least 5 days.

### 2.2. Effect of Anticoagulant Tube, Holding Time and Holding Temperature

After finding conditions for the chemical stabilization of TPN samples, considered for the IEC-ESI-QqQ analysis, other parameters related to the blood collection, RBC isolation, lysis and storage were evaluated with respect to the TPN stability.

**Figure 5:**
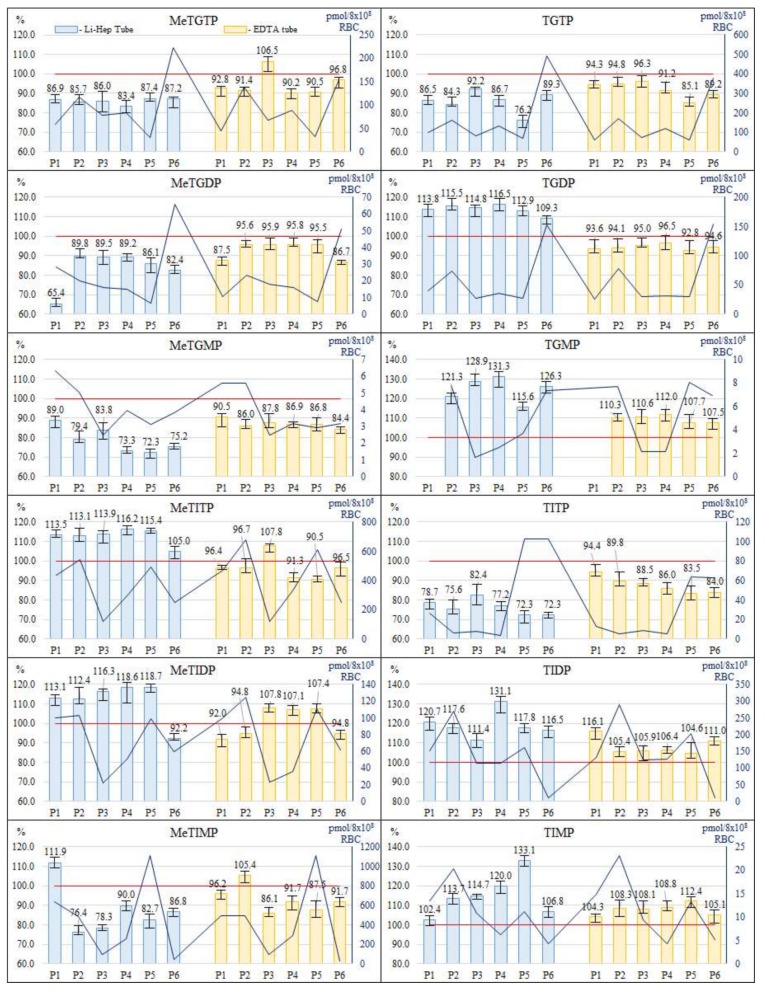
Stability of thiopurine nucleotides in different anticoagulant syringes used for collection of blood samples and different holding times of such samples. Comparison of levels of methylthioguanosine and thioguanosine nucleotides (diagrams in upper half) and methylthioinosine nucleotides and thioinosine nucleotides (diagrams in lower half) in the samples collected into the EDTA or Li-Heparin syringes and hold for 4 h to the samples processed immediately (expressed as %). Dark blue line on the secondary axis represents levels of TPN expressed as pmol/8 × 10^8^ RBC in sample processed immediately.

Type of anticoagulant tube (Li-Heparin vs. EDTA), employed for the collection of blood samples, was studied at the beginning. The samples were taken from 6 patients and collected in the EDTA tube (2 aliquots) and Li-Heparin tube (2 aliquots) from each patient. Both aliquots were treated according to the procedure 3.3 (in saline, RBC lysis and 24 h storage at −20 °C), one aliquot immediately after drawing while another one after 4 h holding at 4 °C. The pre-analytic treatment of the RBC lysates was carried out according to the procedure 3.4 (in PB medium). The effect of both tubes was evaluated by comparing the concentration levels of individual TPN measured in the aliquot holding for 4 h before the RBC isolation to the aliquot processed immediately after drawing. Data from the corresponding IEC-ESI-QqQ measurements are graphically expressed by Figure 5.

An acceptance interval of 90–110% was set for the fluctuations of the TPN concentration levels in the tested anticoagulant syringes. In case of the Li-Heparin tube, 65 of the 71 total measured levels (1 metabolite in one sample was below lower limit of quantification (LLOQ)) were outside of the acceptance interval (i.e., 91.5% outliers ranged in the interval of 65.4–131.3%). Considerably better results were obtained when the samples were collected into the EDTA tubes. In this case, only 22 of the 71 total measured levels were outside of the acceptance criteria (i.e., 31.0% outliers ranged in the interval of 84.0–112.4%) and the fluctuation amplitude was pronouncedly reduced. Based on these experimental findings, the collection of blood samples from the IBD patients into the EDTA tubes and their holding up to 4 h at 4 °C is recommended for the subsequent IEC-ESI-MS/MS measurements of the individual TPN with an acceptable stability (fluctuations of peak areas ca. ±15%).

The interval, used for holding the collected sample before the RBC are isolated (i.e., holding time), varies among the published studies from 4 [27], through 6 [22,24], up to 8 h [26]. Although all of the authors stated that TPN were sufficiently stable within the chosen holding times usually no data were presented to support these statements. In our study the holding time of 4 h and holding temperature 4 °C were chosen as a compromise considering the acceptable stability of the TPN in the samples (experimentally confirmed) and the practical requirements/possibilities of the clinical departments processing such samples (e.g., see clinical affiliations of the authors).

### 2.3. Effect of Solutions Used for RBC Washing and Dilution

Isolation and cleaning procedures for RBC present in biological matrices should be adapted to given analyzed compounds in order to ensure their maximum stability. Although the isolation of RBC is a well-known routine process (in our work generally described in Section 3.3), no experimental verification of the effect of the washing and dilution solutions on the TPN stability has been done so far. Therefore, we evaluated such effect for two different solutions, namely 0.9% NaCl solution (saline) and PBS solution (phosphate-buffered saline). 

**Figure 6:**
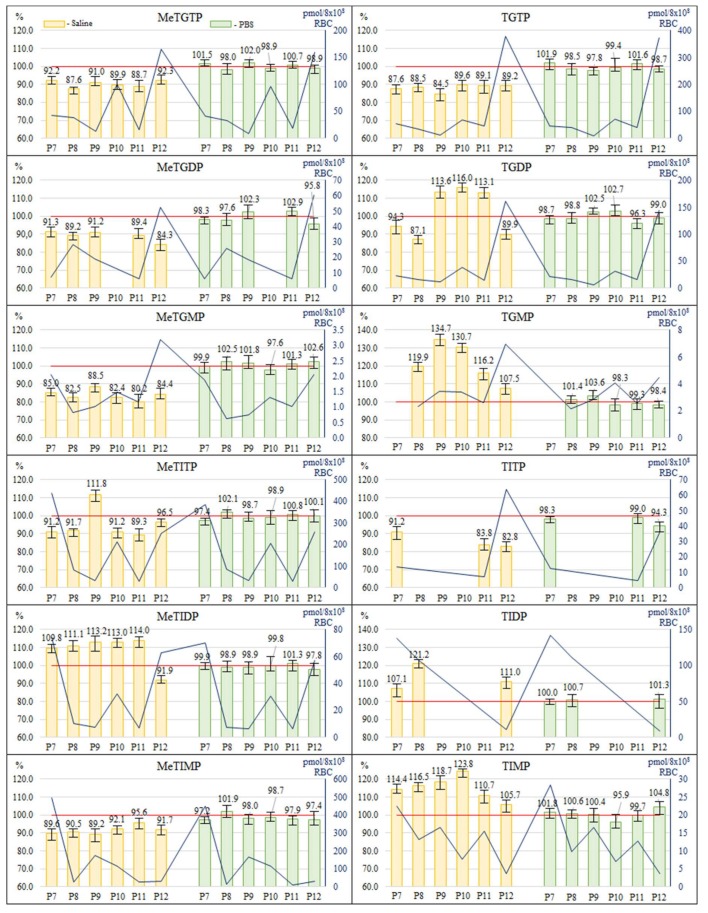
Stability of thiopurine nucleotides in different RBC washing and dilution solutions. Comparison of levels of methylthioguanosine and thioguanosine nucleotides (diagrams in upper half) and methylthioinosine nucleotides and thioinosine nucleotides (diagrams in lower half) in the RBC samples washed and diluted with the saline or PBS and hold for 4 h to the samples processed immediately (expressed as %). Dark blue line on the secondary axis represents levels of TPN expressed as pmol/8 × 10^8^ RBC in sample processed immediately.

The blood samples from 6 patients were collected into the EDTA tubes—four aliquots from each patient. Two aliquots were treated immediately after drawing (Section 3.3, RBC lysis and 24 h storage at −20 °C), in one the RBC were washed and diluted with the saline solution, in another one the RBC were washed and diluted with PBS. The other two aliquots were hold at 4 °C for 4 h and then treated in the same manner as the first two aliquots. The pre-analytic treatment of the RBC lysates was carried out according to the procedure 3.4 (in PB medium). It was found out that some of the results obtained using the different washing and dilution procedures (expressed as the peak areas measured when using PBS divided by the peak areas measured when using saline) significantly differed from each other, with the ratios of the TPN peak areas ranging in the interval of 37.0–132.2%. Here, 52.34% values were out of the acceptance interval (90–110%), i.e., differed significantly from each other. In order to select an appropriate procedure for the protocol, the effect of the washing and dilution solutions was evaluated also by comparing the concentration levels of individual TPN measured in the different aliquots holding for 4 h before the RBC isolation to the aliquots processed immediately after drawing. Data from the corresponding IEC-ESI-QqQ measurements are graphically expressed by Figure 6.

Again the acceptance interval of 90–110% was used. In case of washing and diluting the RBC with the saline solution, 42 of the 64 total measured levels (8 metabolites in several samples were below LLOQ) were outside of the acceptance interval (i.e., 65.6% outliers ranged in the interval of 80.2–134.7%). Considerably better results were obtained when the RBC were washed and diluted with PBS. In this case, none of the 64 total measured levels was outside of the acceptance interval and the all values ranged in the interval of 94.3–104.8%. Based on these experimental findings, the washing and dilution of the RBC with PBS is recommended for the subsequent IEC-ESI-QqQ measurements of the individual TPN levels of IBD patients with an acceptable stability (fluctuations of peak areas ca. ±5%).

### 2.4. Effect of RBC Lysis Temperature and Short-Term Storage Temperature

**Figure 7:**
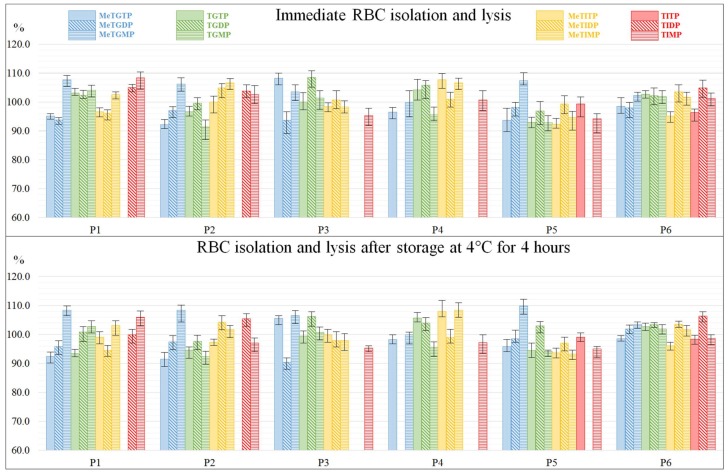
Stability of thiopurine nucleotides under different RBC lysis and short-term storage temperature. The ratios of TPN peak areas referring to the RBC lysed and stored for 24 h at −80 °C and −20 °C are expressed graphically as columns. The RBC lysates were prepared under optimum conditions: (i) EDTA anticoagulant tube for the blood collection; (ii) time between the sample collection and RBC isolation 0 h or 4 h at the temperature of 4 °C (upper and lower diagrams); (iii) phosphate-buffered saline for RBC washing (two-times) and re-suspendation (dilution 1:1); (iv) 50 mmol/L phosphate buffer with pH 7.4 and 10 mmol/L DTT additive as a stabilizing medium for TPN in RBC lysates.

In the final step of the stability study an effect of the RBC lysis and short-term storage temperature on the TPN concentration levels was evaluated. The RBC samples P7–P12, prepared under optimum conditions declared in Section 2.1, Section 2.2 and Section 2.3, were lysed and stored at the defined temperatures (−80 °C and −20 °C) for one day. The results are expressed as the ratio (%) of concentration levels found in lysates prepared and stored at −80 °C to these ones found at −20 °C. All of the quantified values were inside the acceptance interval of 90–110%, as it is documented in Figure 7. Therefore, it was concluded that the RBC lysis and short-term storage temperature did not have any significant effect on the TPN levels measured in the RBC samples of IBD patients. Hence, both temperatures (−20 °C and −80 °C) can be used for the lysis of RBC as well as for the short-term (24 h) storage of the RBC samples intended for the determination of individual TPN by means of the IEC-ESI-QqQ method. 

Although the storage temperature of −80 °C is generally accepted/recommended for biomedical analyses, see e.g., ref. [28,29] for pooled thiopurine samples, the temperature of −20 °C is more economical and, by that, eligible for routine clinical practice. Therefore, currently we are carrying out the long-term stability study to evaluate the effect of different temperature (−80 °C and −20 °C) on the TPN concentration levels present in the RBC samples stored for a long time (from 1 months to several years).

### 2.5. Validation of the LC/MS Method

The calibration curves for each of the TPN were prepared by measuring the calibration solutions (Section 3.2) applying the weighted linear regression (MS Excel 2010). Here, the dependences of the ratio of the analyte peak area to peak area of the internal standard against the concentration of the analyte were measured. The chromatographic elution behavior of the selected mono-, di- or tri-phosphated IS were similar to the mono-, di- or tri-phosphated TPN, respectively. From the practical point of view, a great advantage of the selected IS is also their commercial availability and relatively low cost, especially in comparison to targeted (D or 13C) TPN standards (for the illustration, in case of an individual preparation, a 25 mg amount of one targeted compound can range in the interval of 10.000–20.000 Euros). The LLOQs for all the TPN are represented by their lowest concentrations in the calibration curve for which the response is at least five times the response compared to blank. The concentrations of individual TPN in the patient samples were calculated from the regression lines. The IEC-ESI-QqQ method was validated according to the FDA guideline for Bioanalytical Method Validation [30], part III.B, including: (1) selectivity; (2) accuracy, precision, and recovery; (3) the calibration curve; (4) sensitivity; (5) reproducibility; and (6) stability of analyte in spiked samples. For the performance parameters data, obtained from the calibration and analysis of QC samples, refer to Table 1 and Table 2, respectively.

The IEC-ESI-QqQ method was linear over two decadic orders for all of the analytes with the coefficients of determination higher than 0.9995 (for more details see Table 1). The valuable sensitivity enhancement (in more than one decadic order) for mono-phosphated TPN was due to increasing the analytical signal in the PB medium when comparing to the EDTA medium (see Section 2.1 and Figure 4). Then, the LLOQ values, ranging in the interval of 1–10 nmol/L, were favorable for the quantification of majority of clinical samples taken for this stability study. Hence, it could be effective also for the clinical samples included in TDM of thiopurines. 

The IEC-ESI-QqQ method showed excellent accuracy ranging between 95–105% (for more details see Table 2). Also the intra-day as well as inter-day precision of the method was very good (<10%) showing, among others, the effective stabilization of the analyzed samples. These findings clearly demonstrated the suitability of the proposed protocol (analytical method plus sample preparation) for the highly reliable monitoring of the thiopurine levels in the clinical samples. 

Matrix effects were less pronounced for di- and tri-phosphated TPN (72.1–107.2%) while they were more pronounced for three mono-phosphated TPN (25.0–48.6%). Anyway, these values were even better than those obtained by Hofmann et al. [27], who used the same IEC-ESI-MS/MS method but different sample preparation procedure. When considering the reference sample matrix (EDTA in case of Hofmann et al., PB in our case) we demonstrated more realistic (and better) matrix effects also for mono-phosphated TPN as 50 mmol/L EDTA, causing very strong matrix effect for these analytes, was replaced in our study by PB, causing considerable less pronounced matrix effect (for the influence of EDTA and PB on the analytical signals of mono-phosphated TPN see Section 2.1 and Figure 4). Although the matrix effect can be eliminated by using the calibration in given matrices, still the sample preparation in PB medium is beneficial for better detection response, especially to monophosphates, and, by that, better sensitivity in real clinical assay.

The selectivity of the method, proved by testing the variable RBC matrices, was excellent due to very high orthogonality of the IEC-ESI-QqQ system based on the combination of chromatographic (separation in time) and mass spectrometry (separation in mass) separation principles. No compounds from the biological matrices interfered with the analytes (see illustrative records in Figure 2) so that all 12 TPN could be reliably quantified.

The favorable performance parameters confirmed the suitability of the proposed protocol for its use in routine clinical laboratories aimed at the determination of individual TPN in RBC lysates from IBD patients treated by AZA.

## 3. Materials and Methods

### 3.1. Chemicals and Solutions

Reference standards of twelve TPN, namely MeTGMP, 6-methylthioguanine 5′-diphosphate (MeTGDP), 6-methylthioguanine 5′-triphosphate (MeTGTP), TGMP, TGDP, TGTP, MeTIMP, MeTIDP, 6-methylthioinosine 5′-triphosphate (MeTITP), TIMP, 6-thioinosine 5′-diphosphate (TIDP) and 6-thioinosine 5′-triphosphate (TITP), were obtained from Jena Bioscience (Jena, Germany) at the concentrations of 10 mmol/L. Reference standards of 8-bromoguanosine mono- (BrGMP), di- (BrGDP) and tri-phosphate (BrGTP), used as the internal standards (IS), were also obtained from Jena Bioscience (Jena, Germany). PBS was obtained from Sigma-Aldrich (Steinheim, Germany). DTT, added to the solutions of thiopurines as an antioxidant protecting their thio- moieties from oxidizing to dithio- derivatives, was obtained from AppliChem GmbH (Darmstadt, Germany). Other chemicals were obtained from Fluka Chemie GmbH (Buchs, Switzerland). All chemicals used were of analytical grade. Water used for the preparation of all solutions was demineralized by a Direct-Q 3 UV water purification system (Merck Millipore, Molsheim, France). 

PB solution (50 mmol/L) was prepared by mixing equimolar amounts of Na_2_HPO_4_ and NaH_2_PO_4_ and adjusting the pH to 7.4 with NaOH. EDTA solution (50 mmol/L) was prepared by dissolving the calculated amount of EDTA in demineralized water and adjusting the pH to 10.5 with NaOH. PBS was prepared by dissolving one tablet in 200 mL of demineralized water (as instructed by the manufacturer) what resulted in the solution containing 10 mmol/L PB, 2.7 mmol/L KCl and 137 mmol/L NaCl with pH 7.4. The solutions of DTT were prepared freshly prior to any experiment. The stock solutions of each TPN and IS with the final concentration of 100 µmol/L were prepared in water or PB. The working solutions were prepared by diluting the stock solutions with water or PB. All of the stock solutions were kept frozen at −20 °C until the use.

### 3.2. Calibration Solutions and QC Samples

The calibration solutions were prepared by an appropriate dilution of the standard stock solutions of individual TPN in the concentration range of 0.001–1 µmol/L (8 concentration levels). Each solution contained also IS (BrGMP for mono-phosphated TPN; BrGDP for di-phosphated TPN and BrGTP for tri-phosphated TPN) and RBC lysate matrix (i.e., equimolar mixture of RBC lysates of six healthy volunteers). The RBC lysates were prepared according to the procedures described in the Section 3.3 and Section 3.4. Each of the calibration solution was measured three times. 

The QC samples were prepared at three concentration levels, i.e., 0.01 (low); 0.10 (medium); and 1.00 (high) µmol/L in the RBC lysate matrix (the same as in the calibration solutions) and in the PB matrix (serving as the reference one in the study of matrix effects). Each of the QC samples was measured five times. In the inter-day precision the QC samples were measured during five consecutive days each sample in five replications. 

### 3.3. Procedures for Blood Sampling and RBC Isolation

The blood samples were collected from twelve IBD patients (4 males and 8 females, median age 35 year, range 21–61 years) treated with the standard dose (2 mg/kg, except for one patient TPMT heterozygous with a reduced dose of 1 mg/kg) of AZA at the Department of Gastroenterology of Saint Michael’s Hospital in Bratislava, Slovakia. Blood samples were collected in the prescribed anticoagulant tube (EDTA or Li-Heparin) and immediately placed into the fridge at 4 °C where they were stored until further processing.

The RBC isolation was performed at the Department of Biochemistry and Hematology of Saint Michael’s Hospital. RBC were separated from the whole blood by the centrifugation at 800× *g* for 10 min at 4 °C. After the centrifugation, the plasma, buffy coat and upper layer of RBC were discarded. The isolated RBC were washed twice with the prescribed solution (saline or PBS) and re-suspended (1:1, *v*/*v*—to obtain concentration of approximately 4 × 10^12^ RBC/L) in the same solution. The re-suspended and diluted RBC were counted and then frozen (what resulted in lysis of the RBC) and stored at the prescribed temperature (−20 °C or −80 °C) until further treatment (Section 3.4) and analysis (Section 3.5 and Section 3.6).

The blood samples from the first six patients (P1-P6) were used for the evaluation of anticoagulant tubes (Section 2.2). The blood samples from the rest of the patients (P7–P12) were used for the evaluation of RBC washing solution (Section 2.3). The RBC samples were divided to two sets stored at −20 °C or −80 °C (Section 2.4).

### 3.4. Pre-Analytic Treatment of RBC Lysates

A pre-analytic treatment of the RBC lysates was carried out according to the procedures described in [25], and [26], and in pure demineralized water (as a reference medium). Finally, a modified procedure (related to [26]) with the following parameters was proposed and applied: 160 µL of freshly thawed RBC lysate was mixed with 40 µL of internal standard solution containing 10 µmol/L of BrGTP, BrGDP, and BrGMP, and 15 g/L DTT (serving as a -SH stabilizer) dissolved in 50 mmol/L PB solution (pH 7.4). The mixture was vortex mixed and then 350 µL of cold mixture of methanol and dichloromethane (35:10, *v*/*v*) was added, which resulted in precipitation of proteins present in the sample. After another vortex mixing, the sample was centrifuged at 15,000× *g* for 10 min at 4 °C and the obtained supernatant was filtered through a nylon syringe filter (0.22 µm) and used for the LC-MS analysis.

### 3.5. LC-MS Apparatus

All of the analyses were carried out on the chromatographic apparatus consisting of LC Agilent Infinity System (Agilent Technologies, Santa Clara, CA, USA) equipped with a gradient pump (1290 Bin Pump VL), an automatic injector (1260 HiPals), and column thermostat (1290 TCC). For the evaluation of effect of the stabilizing agents (Section 2.1), the LC system was hyphenated with the quadrupole time-of-flight (Q-TOF) mass spectrometer (Agilent 6520 Accurate-Mass Q-TOF LC/MS) equipped with the electrospray ionization source operated in a positive ionization mode. The Q-TOF mass spectrometer was operated under following parameters: drying gas temperature 325 °C, drying gas flow 12 L/min, nebulizing gas pressure 60 psi, ESI source voltage 4000 V, fragmentor voltage 140 V, collision gas N_2_. For the rest of the analyses (Section 2.2, Section 2.3 and Section 2.4), the LC apparatus was coupled with the triple quadrupole (QqQ) mass spectrometer (Agilent 6410 Triple Quadrupole LC/MS) equipped with the electrospray ionization source operated in a positive ionization mode. The following QqQ parameters were used: drying gas temperature 325 °C, drying gas flow 10 L/min, nebulizing gas pressure 20 psi, ESI source voltage 3500 V, collision gas N_2_. A computer with the Mass Hunter software (version MassHunter Workstation B.05.01) was used for the acquisition and processing of data from the LC-MS apparatus.

### 3.6. Chromatographic Conditions

The chromatographic separation of TPN was carried out according to a properly modified LC-MS method based on the methods used before [27,31]. Briefly, the separation was carried out using an ion-exchange column—Biobasic AX column (2.1 × 50 mm, 5 µm) obtained from Thermo Fisher Scientific (Waltham, MA, USA). The mobile phase A consisted of 10 mmol/L ammonium acetate in 30% acetonitrile, adjusted to pH 6.0 with acetic acid. The mobile phase B consisted of 1 mmol/L ammonium acetate in 30% acetonitrile, adjusted to pH 10.5 with ammonium hydroxide. The following gradient elution was used: 0–1 min—0% B; 1.0–2.5 min—increase to 35% B; 2.5–5.0 min—35% B; 5.0–7.0 min—increase to 65% B; 7.0–10.0 min: 65% B; 10.0–10.5 min—increase to 100% B, 10.5–15.0 min—100% B. The column was reconditioned with the initial composition of the mobile phases (0% B) for 5 min. The flow rate of the mobile phase was set at 0.250 mL/min. In case of the evaluation of stabilizing agents (Section 2.1) the flow rate of 0.500 mL/min was used instead. The gradient of mobile phases was adjusted accordingly. During all of the analyses the column temperature was maintained at 30 °C. The injection volume was 10 μL.

## 4. Conclusions

The updated protocol for the preparation and analysis of the clinical samples related to the thiopurine therapy of IBD was proposed, experimentally verified and recommended for the routine clinical use. This comprehensive stability study indicated the optimum parameters for the blood collection, RBC preparation and storage, i.e., the parameters playing a crucial role in the thiopurine stability during the initial handling with biological material at the clinical department. It is concluded that the following parameters of the sample preparation are prerequisite for the IEC-ESI-QqQ measurements of the profiles of 12 phosphated thiopurine metabolites present in blood samples taken from IBD patients treated by azathioprine, ensuring an acceptable stability of the analytes: (i) EDTA anticoagulant tube for the blood collection; (ii) time between the sample collection and RBC isolation up to 4 h at the temperature of 4 °C; (iii) phosphate-buffered saline for RBC washing (two-times) and re-suspension (dilution 1:1); (iv) temperature of −20 °C for the RBC lysis and short-term storage (up to 24 h); (v) 50 mmol/L phosphate buffer with pH 7.4 and 10 mmol/L DTT additive as a stabilizing medium for TPN in RBC lysates.

Implementation of this updated protocol, combining the well-defined sample preparation with advanced analysis, can help reduce the discrepancies in future clinical studies highly needed to redefine the role of thiopurines therapeutic drug monitoring in the optimization of IBD therapy. An importance of the proposed protocol for clinicians is amplified by the recent findings demonstrating a dose-dependent synergetic pharmacokinetic effect of thiopurines used in IBD treatment when combined with biological drugs (e.g., monoclonal antibodies such as infliximab) [32].

## Figures and Tables

**Table 1:** Performance parameters of the IEC-MS/MS method for determination of twelve thiopurine nucleotides in spiked RBC lysate matrix ^a^.

	Linearity Range (µmol/L)	r^2^	Slope (b)	SD_b_	Intercept (a)	SD_a_	LLOQ (µmol/L)
**MeTGTP**	0.010–1	0.9995	1.9440	0.0108	0.0005	0.0049	0.010
**MeTGDP**	0.005–1	0.9997	4.0207	0.0150	0.0005	0.0064	0.005
**MeTGMP**	0.005–1	0.9998	18.8268	0.0548	−0.0003	0.0233	0.005
**TGTP**	0.010–1	0.9998	2.1550	0.0066	0.0006	0.0031	0.010
**TGDP**	0.005–1	0.9996	2.8510	0.0126	−0.0005	0.0053	0.005
**TGMP**	0.005–1	0.9998	5.3872	0.0180	−0.0002	0.0076	0.005
**MeTITP**	0.010–1	0.9995	5.4325	0.0298	0.0008	0.0137	0.010
**MeTIDP**	0.001–1	0.9997	23.0756	0.0784	0.0006	0.0312	0.001
**MeTIMP**	0.005–1	0.9999	9.0761	0.0200	0.0004	0.0085	0.005
**TITP**	0.010–1	0.9997	3.6514	0.0162	−0.0003	0.0074	0.010
**TIDP**	0.005–1	0.9998	3.2178	0.0097	0.0001	0.0041	0.005
**TIMP**	0.005–1	0.9998	9.7267	0.0314	0.0005	0.0133	0.005

^a^ For the preparation of calibration solutions see Section 3.2. For the working conditions of the IEC-ESI-MS/MS method see Section 3.6.

**Table 2:** Analysis of QC samples by IEC-MS/MS method ^a^.

	QC Low				QC Medium				QC High			
	Accuracy (%)	Intra-Day Precision RSD (%)	Inter-Day Precision RSD (%)	Matrix Effects (%)	Accuracy (%)	Intra-Day Precision RSD (%)	Inter-Day Precision RSD (%)	Matrix Effects (%)	Accuracy (%)	Intra-Day Precision RSD (%)	Inter-Day Precision RSD (%)	Matrix Effects (%)
**MeTGTP**	98.03	2.74	6.79	86.21	102.23	1.59	4.89	90.55	100.20	2.09	4.83	90.98
**MeTGDP**	96.85	2.20	5.72	104.83	98.89	2.17	4.20	105.90	105.76	1.62	5.92	107.20
**MeTGMP**	97.17	2.27	4.67	27.61	97.11	3.38	4.57	29.71	100.80	2.12	3.35	30.04
**TGTP**	95.68	3.12	4.01	72.13	104.42	4.11	6.14	79.53	99.56	1.17	2.44	76.32
**TGDP**	95.33	2.24	5.23	100.22	99.55	1.89	3.09	100.43	102.49	2.05	3.89	102.54
**TGMP**	94.22	2.59	4.35	46.24	96.85	4.91	5.27	48.62	102.61	2.13	3.46	45.31
**MeTITP**	95.63	2.27	5.09	90.42	101.53	1.44	4.49	92.08	103.17	1.68	4.87	93.99
**MeTIDP**	97.08	2.98	5.80	79.87	99.39	2.87	4.54	78.86	104.82	2.48	5.05	79.32
**MeTIMP**	96.76	3.99	4.33	24.98	98.54	3.55	4.47	26.55	100.74	1.00	3.97	25.94
**TITP**	95.34	1.90	3.53	75.02	103.13	2.38	5.83	77.73	102.92	1.47	3.85	77.12
**TIDP**	96.89	2.38	4.75	98.96	99.53	2.78	5.57	96.98	105.73	1.97	4.78	97.91
**TIMP**	96.02	3.31	4.02	101.78	97.51	2.98	5.01	106.52	102.58	2.02	2.80	104.39

^a^ For the preparation of QC samples see Section 3.2. For the working conditions of the IEC-ESI-MS/MS method see Section 3.6.
